# Research Progress of UiO-66-Based Electrochemical Biosensors

**DOI:** 10.3389/fchem.2022.842894

**Published:** 2022-01-26

**Authors:** Ming Wu, Qi Zhang, Qiuyu Zhang, Huan Wang, Fawei Wang, Junmei Liu, Liquan Guo, Kai Song

**Affiliations:** ^1^ Key Laboratory of Straw Comprehensive Utilization and Black Soil Conservation, Ministry of Education, College of Life Science, Jilin Agricultural University, Changchun, China; ^2^ College of Food Science and Engineering, Jilin Agricultural University, Changchun, China; ^3^ College of Life Sciences, Engineering Research Center of the Chinese Ministry of Education for Bioreactor and Pharmaceutical Development, Jilin Agricultural University, Changchun, China; ^4^ School of Life Science, Changchun Normal University, Changchun, China

**Keywords:** UiO-66, electrochemical biosensor, environmental, food safety, biomedical

## Abstract

UiO-66, as a member of the MOFs families, is widely employed in sensing, drug release, separation, and adsorption due to its large specific surface area, uniform pore size, easy functionalization, and excellent stability. Especially in electrochemical biosensors, UiO-66 has demonstrated excellent adsorption capacity and response signal, which significantly improves the sensitivity and specificity of detection. However, the existing application research remains in its infancy, lacking systematic methods, and recycling utilization and exclusive sensing of UiO-66 still require further improvement. Therefore, one of the present research objectives is to explore the breakthrough point of existing technologies and optimize the performance of UiO-66-based electrochemical biosensors (UiO-66-EBs). In this work, we summarized current experimental methods and detection mechanisms of UiO-66-EBs in environmental detection, food safety, and disease diagnosis, analyzed the existing problems, and proposed some suggestions to provide new ideas for future research.

## 1 Introduction

Metal-organic frameworks (MOFs) have garnered considerable attention due to their structural diversity, large specific surface area, and high porosity (Liu et al., 2021). Their applications cover a wide range of fields from environment, medical treatment, biology, energy, and electronics. However, the processability and conductivity of most MOFs are suboptimal, and their structural stability decreases with increasing the length of organic ligands (Zou and Liu, 2019). To circumvent this issue Cavka et al., 2008, synthesized rigid MOFs named UiO-66 using Zr as the metal center and terephthalic acid as the organic ligand (Cavka et al., 2008). Due to its uniform adjustable, high specific surface area, strong Zr-O bonds and higher ZR (IV) coordination numbers, the chemical stability of UiO-66 is given (Piscopo et al., 2015). More importantly, the unsaturated coordination of Zr in the structure produces L-acidic sites (Valenzano et al., 2011), which are usually used as catalytic active centers or carriers for loading catalytically active components (Abánades Lázaro and Forgan, 2019). Meanwhile, the developed microporous structure of UiO-66 can selectively adsorb specific substances or produce a crystal fluorescence effect by group modification (Abánades Lázaro and Forgan, 2019). These properties facilitate the preparation of high-performance UiO-66-EBs, ultimately achieve selective identification for specific substances, and associated with the concentration change of the quenched components.

**FIGURE 1:**
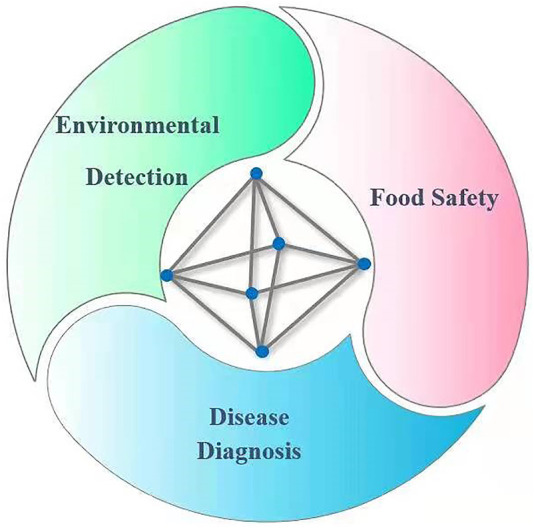
Table of contents graphic.

Although there are various methods for the synthesis of UiO-66, including microwave, microfluidic and continuous flow methods, mechanochemical, evaporation, and electrochemical synthesis, the most important one is the solvothermal method (Zhang et al., 2020). The harsh reaction conditions, costly raw materials, the selectivity and recyclability of UiO-66-EBs in practical applications, as well as sensitivity and proprietary sensing, are critical factors to consider. This paper summarizes the design and performance of UiO-66-EBs (as shown in Table 1) in three areas: environmental detection, food safety, and disease diagnosis to provide practical ideas and theoretical references for developing simple, portable, real-time and efficient UiO-66-EBs (Figure 1).

**TABLE 1:** Performance of UiO-66-EBs.

Detection method	Sample	Linear range	Detection limit	References
Fluorescence spectrum	Cysteine and glutathione	10^−11^–10^−3^ M	10^−11^ M	Li et al. (2015)
Fluorescence resonance energy transfer	Mercury	0.1–10 mM	17.6 nM	Wu et al. (2016)
Linear sweep voltammetry	Telomerase	5 × 10^2^–10^7^ Hela cells/ml	100 Hela cells/ml	Ling et al. (2016)
Fluorescence spectrum	H_2_S	0–10 mM	6.46 μM	Li et al. (2017)
Electrochemical impedance spectroscopy	Hydroquinone, catechol and resorcinol	0.5–100 μM, 0.4–100 μM and 30–400 μM	0.056, 0.072 and 3.51 μM	Deng et al. (2017)
Square wave voltammetry	Kanamycin and chloramphenicol	0.002–100 nM	0.16 and 0.19 pM	Chen et al. (2017)
Electrochemical impedance spectroscopy and differential	Carcinoembryonic antigen	0.01–10 ng/ml	8.88 and 4.93 pg/ml	Guo et al. (2017)
Electrochemical impedance spectroscopy	PKA	0.005–50 μ/ml	0.0049 μ/ml	Wang et al. (2017)
Electrochemical impedance spectroscopy	Diethylstilbestrol	0.1 pg/ml–20 ng/ml	0.06 pg/ml	Wu et al. (2018)
Fluorescence measurements	ATP	0–1 μM	35 nM	Xu and Liao (2018)
Differential pulse voltammetry indicated	The *mycobacterium tuberculosis* antigen MPT64	0.02–1,000 pg/ml	10 fg/ml	Li et al. (2018)
Electrochemical impedance spectroscopy	PKA	0.015–80 μ/ml	0.009 μ/ml	Yan et al. (2018)
Voltammetry	Organophosphorus compounds	0.01–150 nM	0.004 nM	Mahmoudi et al. (2019)
Electrochemical impedance spectroscopy and differential pulse voltammetry	Patulin	5 × 10^−8^–5 × 10^−1^ μg/ml	1.46 × 10^−8^ μg/ml	He and Dong (2019)
Cyclic voltammetry and electrochemical impedance spectroscopy	Prostate specific antigen	0.0001–10 ng/ml	0.038 pg/ml	Fang et al. (2019)
Combining of amperometric and square wave voltametric methods	Amyloid β-protein	10 fg/ml–100 ng/ml	3.35 fg/ml	Miao et al. (2019)
The obtained electrochemical impedance	The cancer cell	1.0 × 10^2^–1.0 × 10^6^ cells/ml	90 cells/ml	Du et al. (2019)
Differential pulse voltammetry indicated	Let-7a and microRNA-21	0.01–10 pM and 0.02–10 pM	3.6 and 8.2 fM	Chang et al. (2019)
Cyclic voltammetry and electrochemical impedance spectroscopy	Breast cancer biomarker CA15-3	5 × 10^−4^–5 × 10^2^ μ/ml	1.7705 × 10^−5^ μ/ml	Xiong et al. (2019)
Electrochemical impedance spectroscopy measurements	N^6^-methyladenosine	0.05–30 nM	0.0167 nM	Wang et al. (2019)
Differential pulse voltammetry indicated	Cardiac troponin I	0.01–100 ng/ml	5.7 pg/ml	Luo et al. (2019)
Cyclic voltammetry and square wave voltammetry	Antibiotics	25–900 nM	13 nM	Yao et al. (2020)
Cyclic voltammetry and electrochemical impedance spectroscopy	Ochratoxin A	0.1 fM–2.0 μM	0.079 fM	Qiu et al. (2020)
Cyclic voltammetry	Low density lipoprotein	1.0 ng/ml–100 μg/ml	0.3 ng/ml	Wang et al. (2020)
Cyclic voltammetry and differential pulse voltammetry	MicroRNA-21	20 fM–600 pM	0.713 fM	Meng et al. (2020)
Electrochemical impedance spectroscopy measurements	β-amyloid	10^−5^–50 ng/ml	3.32 fg/ml	Dong et al. (2020)
Electrochemical impedance spectroscopy measurements	Osteopontin	0.01 pg/ml–2.0 ng/ml	4.76 fg/ml	Zhou et al. (2020)
Cyclic voltammetry and Electrochemical impedance spectroscopy	NT-proBNP	1 fg/ml–100 ng/ml	0.41 fg/ml	Wang et al. (2020)
Cyclic voltammetry and electrochemical impedance spectroscopy	Living Michigan cancer foundation-7 cancer cells	100–100,000 cell/ml	31 cell/ml	Li et al. (2020)
Electrochemical impedance spectroscopy	CEA	50 fg/ml–10 ng/ml	16 fg/ml	Bao et al. (2020)
Ratiometric fluorescent method	Detection of dopamine and reduced glutathion	4–50 μM and 1–70 μM	0.68 and 0.57 μM	Wang et al. (2020)
Cyclic voltammetry, differential pulse voltammetry and electrochemical impedance spectroscopy	Tetracycline	1.0 × 10^−6^–6.0 × 10^−5^ mol/L	8.94 × 10^−7^ mol/L	Zhong et al. (2021)
Electrochemical impedance spectroscopy and cyclic voltammetry	Nitrogenous diphenyl ether pesticide	0–100 μM	0.026 μM	Cheng et al. (2021)
Square wave voltametric methods and electrochemical impedance spectroscopy	*Staphylococcus aurens*	10–10^9^ cfu/ml	3 cfu/ml	Wang et al. (2021)
Colorimetric and spectrofluorometric techniques	Cholesterol quantification	0.04–1.60 μmol/L	0.01 μmol/L	Abdolmohammad-Zadeh et al. (2021)
Photoelectrochemical and electrochemical tests	PKA	0.001–100 μ/ml	0.00035 μ/ml	Xiao et al. (2021)
Fluorescence spectra	Bacterial	2.5 × 10^4^–5.0 × 10^4^ CFU/ml	1.0 CFU/ml	Zuo et al. (2021)
Resistance method	Exosomes-derived	1.0 × 10^3^–1.0 × 10^8^ Particles/ml	300 Particles/ml	Gu et al. (2021)
Differential pulse voltammetry indicated	Procalcitonin	1 pg/ml–100 ng/ml	0.3 pg/ml	Miao et al. (2021)
Linear sweep voltammetry	Alpha-fetoprotein	1 fg/ml–100 ng/ml	0.2 fg/ml	Ding et al. (2020)
Electrochemical impedance spectroscopy	ATP	1.0 × 10^−5^–5.0 ng/ml	1.69 fg/ml	Zhu et al. (2020)
Cyclic voltammetry	Glucose	1–10 mM	5 μM	Jin et al. (2021)
Electrochemical impedance spectroscopy and cyclic voltammetry	ATP	1.0 × 10^−5^–5.0 ng/ml	5.04 fg/ml	Zhang et al. (2021)

## 2 Application of UiO-66-Based Electrochemical Biosensors

### 2.1 Environmental Detection

UiO-66-EBs utilized in environmental applications are often designed to detect toxic substances by monitoring changes in fluorescence and conductivity.

In 2016, Wu et al. anchored thymine (T)-rich ssDNA to an aromatic organic linker on UiO-66-NH_2_ amino functional group via π-π stacking and hydrogen bonding. Due to light-induced energy transfer, fluorophore (FAM) fluorescence-labeled at ssDNA 3 end was effectively suppressed. While in the presence of Hg^2+^, T-Hg^2+^-T interaction disrupts the hybrid structure of ssDNA and UiO-66-NH_2_, and FAM fluorescence is restored, allowing for Hg^2+^ detection (Wu et al., 2016). Li et al. also fully exploited the feature of H_2_S to reducibly destroy the rigid surface of C=C double bond and fluorescence conjugation in UiO-66-CH = CH_2_ structure (Li et al., 2017). Compared with the aforementioned method, the ingenious introduction of vinyl groups simplifies the detection steps and avoids the instability of the hybrid system. Deng et al. prepared a large pore size zirconium-based mesoporous carbon (MC) composite (UiO-66/MC) using the hydrothermal method (Arduini et al., 2016; Deng et al., 2017), allowing for rapid electron transfer and promoting mass transfer for co-detection of dihydroxybenzene isomers (DBIs) of hydroquinone (HQ), catechol (CT) and resorcinol (RS) with detection limits of 0.056, 0.072, and 3.51 μM, respectively (Deng et al., 2017). To further improve the sensitivity of UiO-66-EBs, Mahmoudi et al. combined metal Ce with multi-walled carbon nanotubes (MWCNTs) into UiO-66 carriers (Mahmoudi et al., 2019), which enhanced the bioaffinity (Guo et al., 2014), conductivity, and signal strength of the sensor due to oxygenophilic and redox properties of Ce and excellent conductivity and catalytic properties of MWCNTs (Xin et al., 2012). The detection limit of biosensor can be as low as 0.004 nM for organophosphorus compounds (Mahmoudi et al., 2019).

As can be observed, the sensitivity of UiO-66-EBs have reached an acceptable detection level in complex environments. In addition to further improving the detection process, future research should also avoid secondary contamination.

### 2.2 Food Safety

UiO-66-EBs mainly use the high selectivity of aptamers to targets and specific recognition principle of enzymes and antibodies to detect foodborne pathogenic bacteria, antibiotics, etc.

In 2017, Chen et al. employed UiO-66-NH_2_ as a carrier to co-immobilize metal ions (Pb^2+^ or Cd^2+^) and cDNA as signal tags for detecting kanamycin and chloramphenicol, respectively. In addition to enhancing the sensor’s stability and electron transfer rate, this signal tag can also realize signal amplification (Chen et al., 2017). Based on that, the electrode was modified with the nano-hybrid product UiO-66-NH_2_/MCA/MWCNT@rGONR. Since the composite material is rich in amino groups and active sites, cDNA strand containing amino groups at the end of nucleic acid aptamer can not only be fixed on its surface but also be embedded within it through superposition and electrostatic interactions between the organic framework and cDNA strand, allowing the aptamer to be more firmly bound to it and avoiding inaccurate detection results caused by the aptamer falling off. The sensor detects kanamycin in the range of 25–90 nM with a detection limit of 13 nM; the average spiked recoveries were 98.3∼107.7% and 97.8∼103.7% with relative standard deviations of 2.01∼4.86% for fish and milk samples, respectively (Yao et al., 2020). He et al. prepared an aminated multilayer metal-organic backbone HP-UiO-66-NH_2_, which provided more active sites for electrochemical detection medium methylene blue and aptamer. The sensor uses MOF@MB-Apt as a signal tag, which is bound to cDNA on the surface of nanoflower-modified electrode of AuNPs-CS-ZnO (He and Dong, 2019). However, because electroactive molecules such as methylene blue are single-point labeled to the aptamer in this construction, the sensitivity and signal output are reduced. As a result, Qiu et al. constructed a double-labeled sequence electrochemical biosensor utilizing a strong Zr-O-P coordination bond in conjunction with the addition of, UiO-66 to PO_4_^3−^ end group DNA carrying OTA. The sensor exhibits high target recognition capability and does not require sophisticated pre-processing (Qiu et al., 2020).

Different from aptamer recognition electrochemical sensors, enzyme-catalyzed UiO-66-EB possess ultra-high sensitivity. Zhong et al. developed MCS@UiO-66-NH_2_/Lac biorecognition element using UiO-66-NH_2_ and mesoporous carbon spheres (MCS), with the advantage of protecting the laccase activity while also improving the stability and conductivity of enzyme-modified electrodes (Zhong et al., 2021). Besides, Cheng et al. immobilized *Pseudomonas aeruginosa* lipase with UiO-66 and proline-modified UiO-66 as carriers to prepare nitrophenol biosensor. Due to the addition of proline, allosteric activation changed the conformation of enzyme, increasing its catalytic activity and improving the sensor’s electrochemical performance (Cheng et al., 2021). Due to high requirements for immobilized materials in terms of pH and temperature, enzymes are easily inactivated after long-term storage. Wang et al. replaced the enzyme-catalyzed reaction with antigen-antibody binding, and they immobilized the yolk antibody on the electrode and combined it with UiO-66 electrochemical signal tag covalently linked ferrocene and phenylboronic acid. The sensor can detect *S. aureus* in the range of 10–109 cfu/ml, with a low detection limit of 3 cfu/ml and a detection time of 20 min (Wang et al., 2021).

Most UiO-66-EBs for food safety detection are designed to enhance the electron transfer rate of the carrier by adsorbing metal ions, or providing more active sites for the airline, as well as to improve sensitivity using enzyme catalysis and antigen-antibody specific recognition. In the future, while we can continue to improve the conductivity and adsorption capacity of UiO-66, we may also focus on signal conversion efficiency and detection process simplification.

### 2.3 Disease Diagnosis

In the field of disease diagnosis, researchers constantly optimize the structure of UiO-66-EBs carrier materials, the composition of signal probes and the detection process to identify more cost-effective and applicable detection methods.

#### 2.3.1 Electrochemical Biosensor Using UiO-66 as a Carrier

In 2015, Li et al. constructed the first Mi-UiO-66 and Mi-UiO-67-based fluorescent probes to detect cysteine and glutathione in living cells. Compared with conventional organic searches, it has better water solubility and does not accumulate in water and cause an explosion and cell damage (Li et al., 2015). With the introduction of fluorescence technology, polydopamine underwent structural modification (Xu and Liao, 2018), becoming rich in Ru (bpy)_3_^2+^ as signal amplification elements (Wang et al., 2017, Wang et al., 2019) and UiO-66-EBs based on fluorescence resonance energy transfer (FRET) (Wang et al., 2020a) was also created. Using high-temperature calcination, Xiao’s group prepared UiO-66-based ZrO_2_ octahedral adsorbed CdS nanoparticles. In the presence of ATP, ZrO_2_/CdS structure binds to a protein kinase A (PKA)-specific peptide on the electrode, and PKA activity is detected by light, without pretreatment or noise (Xiao et al., 2021).

Numerous UiO-66-based nanocomposites have been extensively developed to enhance signal intensity. Du et al. pioneered the preparation of electrochemical impedance biosensors using high specific surface area and porosity of the organic framework of folate-functionalized zirconium metal (Wang et al., 2019). Inspired by this, several sensors have emerged, including bio-impedance sensors using ZrO_2_@GNF nanohybrids composed of high-temperature calcined polyacrylonitrile-coated UiO-66 as carriers (Zhou et al., 2020), composite probe sensors composed of UiO-66 adsorption aptamers, and ferrocene (Fc) as signal tags (Wang et al., 2020b), and sensors based on Pd@UiO-66 nanocomposites (Meng et al., 2020). To further improve the detection efficiency, Miao et al. enhanced the sensor’s electrochemical response signal by employing UiO-66 as a carrier to adsorb a large amount of toluidine blue (Miao et al., 2021). Hossein’s group developed a kind of fluorescent biosensor using the principle of enzymatic oxidation to modulate the photocatalytic activity of GQDs/UiO-66 nanocomposites, simplifying the process while also improving sensitivity (Abdolmohammad-Zadeh et al., 2021). Zuo et al. proposed a more simplistic fluorescent free labeled sensor based on Zr-UiO-66-B(OH)_2_ nanocomposite as a carrier for efficient bacterial monitoring and inactivation (Zuo et al., 2021).

The introduction of a bimetallic organic framework enriches the sensor design. In 2018, Yan et al. proposed Au&Pt@UiO-66 to detect PKA activity and inhibitor screening (Yan et al., 2018). UiO-66 as a carrier inhibited metal nanoparticle aggregation. Due to the synergistic impact, the bimetallic nanoparticles outperformed the monometallic nanoparticles in terms of catalytic activity, enhancing the electrochemiluminescence signal of the sensor. Subsequently, Miao et al. implemented further improvements based on bimetallic nanoparticles and developed a Cu-Al_2_O_3_-g-C_3_N_4_-Pd and UiO-66@PANI-MB-based dual signal sandwich electrochemical immunosensor for amyloid (Aβ) detection. The sensor also utilizes a square wave voltammetry signal while using a current ampere I-t curve signal.UiO-66@PANI-MB as a signal tag compared to UiO-66 helps stabilize electrode structure and increase electron transfer rate. The dual-signal mode improves the analytical performance of electrochemical immunosensor and is vital for the prediagnosis of Alzheimer’s disease (Miao et al., 2019).

AgNCs@Apt@UiO-66-based electrochemical biosensors were also developed and employed for carcinoembryonic antigen (CEA) detection. The composite combines the advantages of each component with high specific surface area, good water stability, low toxicity, and specificity, exhibiting high biocompatibility and electrochemical properties. It does not require complex sample pretreatment and is suitable for detecting human serum samples (Guo et al., 2017). In addition, Luo’s group constructed a voltammetric sensor to analyze cardiac troponin I using a double aptamer, the core of which is composed of a DNA nanotetrahedron-connected double aptamer and a magnetic metal-organic backbone Fe_3_O_4_@UiO-66 (Luo et al., 2019).

#### 2.3.2 Electrochemical Biosensor Using UiO-66-NH_2_ as a Carrier

Due to the protonization and unique microporous structure of -NH_2_ in UiO-66-NH_2_, the UiO-66-NH_2_ ratio UiO-66 has more surface negative potentials, enhances selectivity to cationic dye adsorption. Typical examples include the introduction of UiO-66-NH_2_ loaded with more electroactive dyes and luminescent reagents Ru (bpy)_3_^2+^; the former prepared functionalized MOFs for simultaneous detection of let-7a and miRNA-21 (Chang et al., 2019), whereas the latter employed the fluorescence quenching principle to detect CA15-3 (Xiong et al., 2019). Subsequently, Dong et al. constructed an electrochemiluminescent immunosensor based on a bimetallic-organic framework composed of UiO-66-NH_2_ and MIL-101. The high porosity and large functional groups of the bimetallic-organic framework improved the carrier loading and binding rate of biomolecules (Dong et al., 2020). Enlightened by this, Gu et al. developed a self-powered biosensor, which optimized the enzyme’s stability and the electroactive probe’s sensitivity to detect exosomes from cancer cells, witn a detection limit of 300 targets per mL (Gu et al., 2021). To further improve detection efficiency and sensitivity of CEA, Bao’s group deployed DNA-gated UiO-66-NH_2_ as a nanocarrier loaded with methylene blue to demonstrate three-dimensional biosensing trajectory of detector by cascade amplification of detection signal (Bao et al., 2020). Li et al. further optimized the carrier material and utilized UiO-66-2NH_2_ adsorbed aptamer (PO_4_-Apt) to detect live breast cancer (MCF-7) cells. In addition to having more amino groups than UiO-66-NH_2_, the complexity and diversity of UiO-66-2NH_2_ could also help improve the stability of the aptamer binding to the cellular complex (Li et al., 2020).

Additionally, the metal-modified UiO-66-NH_2_ extends the sensor design. Ling’s group designed a biosensor to detect multicellular telomerase activity using UiO-66-NH_2_ adsorbed platinum nanoparticles. The method is easy to operate, does not require additional separation steps, and allows other signal amplification to be easily integrated (Ling et al., 2016). Wu et al. first employed Au/UiO-66-NH_2_/CdS nanocomposite as a photoactive matrix to improve electron transfer rate, photoelectric conversion efficiency, and sensor’s selectivity by modification with Au and CdS nanoparticles. (Wu et al., 2018). Subsequently, Fang et al. designed a sandwich electrochemiluminescence immunosensor using Ag^+^@UiO-66-NH_2_@CdWS. The water stability of UiO-66-NH_2_ itself, and the modification of metal ions, provided more binding sites for the luminescent carrier (Fang et al., 2019). Wang and Ding et al. respectively extended the above design by developing an electrochemiluminescent biosensor for detecting amino-terminal precursor peptide of brain natriuretic peptide (NT-proBNP) using UiO-66-NH_2_ as a template (Wang et al., 2020), by forming a magnetic metal-organic backbone to immobilize CdSnS nanocrystals by further growth on UiO-66-NH_2_ structure (Ding et al., 2020). Jin et al. went a step further by immobilizing glucose oxidase (GOx) on ruthenium-based conjugated polymers and UiO-66-NH_2_ nanocomposites, avoiding the weak electronic conductivity of MOFs and increasing the biocompatibility and stability. This design opens a new path for applying enzyme electrochemical biosensors and enzyme biofuel cells (EBFCs) (Jin et al., 2021).

Aptamer-based electrochemical biosensors based on UiO-66-NH_2_ are also a hot research topic. Li et al. employed UiO-66-NH_2_ as a carrier for detecting *Mycobacterium tuberculosis* antigen MPT64 in serum by immobilizing gold nanoparticles, aptamer, and horseradish peroxidase as signal probes (Li et al., 2018). To improve the sensor’s recognition performance, Zhu et al. combined graphene oxide and UiO-66 to enhance the aptamer’s affinity to the carrier (Zhu et al., 2020). In addition, Zhang et al. successfully prepared an aptamer electrochemical sensor based on core-shell UiO-66-NH_2_@COF composite to detect ATP and chloramphenicol using the covalent coupling method (Zhang et al., 2021).

It is easy to observe a diverse range of sensors in the field of disease diagnosis using UiO-66/UiO-66-NH_2_ nanocomposites as carriers. The nanocomposites retain and maximize the benefits of each component, thus improving sensor performance. The future goal remains the development of UiO-66-like structures and the search for more superior functionalized MOFs and nanocomposites with increased active sites and stability as sensing platforms.

## 3 Conclusion and Outlook

UiO-66 is widely used in electrochemical biosensors due to its excellent adsorption capacity, ease of functionalization, and high specific surface area. Moving from UiO-66 to UiO-66-NH_2_ and then to their nanocomposites, the sensor’s bioaffinity, electron transfer rate, and electrical conductivity have been improved. However, current UiO-66-EBs still have much room for improvement in terms of response sensitivity and electrochemical performance. For example, a sensor of binding enzymes still needs to find a more excellent mandatory enzyme, and maximize the activity of maintaining enzymes, making it more functional characteristics. Future development efforts should be directed at optimizing the structure and stability of UiO-66-based carrier materials, increasing their functional characteristics. Additionally, to compensate for the low detection sensitivity of UiO-66 nanomaterials due to their low electrical conductivity, the next stage of research should focus on exploring the comprehensive performance of UiO-66 complexes modified with different metal ions or functional groups and synthesizing more valuable UiO-66 nanohybrids to be introduced into electrochemical biosensors, to enhance their practical applicability further. Meanwhile, the future development trend of UiO-66-EBs and its derivatives should be more green, multifunctional, and industrialized.
